# What is new in FungiDB: a web-based bioinformatics platform for omics-scale data analysis for fungal and oomycete species

**DOI:** 10.1093/genetics/iyae035

**Published:** 2024-03-26

**Authors:** Evelina Y Basenko, Achchuthan Shanmugasundram, Ulrike Böhme, David Starns, Paul A Wilkinson, Helen R Davison, Kathryn Crouch, Gareth Maslen, Omar S Harb, Beatrice Amos, Mary Ann McDowell, Jessica C Kissinger, David S Roos, Andrew Jones

**Affiliations:** Institute of Systems, Molecular and Integrative Biology, University of Liverpool, Liverpool L69 7BE, UK; Institute of Systems, Molecular and Integrative Biology, University of Liverpool, Liverpool L69 7BE, UK; Genomics England Limited, London E14 5AB, UK; Institute of Systems, Molecular and Integrative Biology, University of Liverpool, Liverpool L69 7BE, UK; Institute of Systems, Molecular and Integrative Biology, University of Liverpool, Liverpool L69 7BE, UK; Institute of Systems, Molecular and Integrative Biology, University of Liverpool, Liverpool L69 7BE, UK; Institute of Systems, Molecular and Integrative Biology, University of Liverpool, Liverpool L69 7BE, UK; School of Infection and Immunity, University of Glasgow, Glasgow G12 8QQ, UK; Imperial College London, London SW7 2BU, UK; University of Pennsylvania, Philadelphia, PA 19104, USA; Wellcome Genome Campus, Hinxton CB10 1SA, UK; University of Notre Dame, Notre Dame, IN 46556, USA; University of Georgia, Athens, GA 30602, USA; University of Pennsylvania, Philadelphia, PA 19104, USA; Institute of Systems, Molecular and Integrative Biology, University of Liverpool, Liverpool L69 7BE, UK

**Keywords:** bioinformatics, database, omics, fungi, oomycetes, Galaxy, Apollo, search strategy, data mining, web-based resource

## Abstract

FungiDB (https://fungidb.org) serves as a valuable online resource that seamlessly integrates genomic and related large-scale data for a wide range of fungal and oomycete species. As an integral part of the VEuPathDB Bioinformatics Resource Center (https://veupathdb.org), FungiDB continually integrates both published and unpublished data addressing various aspects of fungal biology. Established in early 2011, the database has evolved to support 674 datasets. The datasets include over 300 genomes spanning various taxa (e.g. Ascomycota, Basidiomycota, Blastocladiomycota, Chytridiomycota, Mucoromycota, as well as Albuginales, Peronosporales, Pythiales, and Saprolegniales). In addition to genomic assemblies and annotation, over 300 extra datasets encompassing diverse information, such as expression and variation data, are also available. The resource also provides an intuitive web-based interface, facilitating comprehensive approaches to data mining and visualization. Users can test their hypotheses and navigate through omics-scale datasets using a built-in search strategy system. Moreover, FungiDB offers capabilities for private data analysis via the integrated VEuPathDB Galaxy platform. FungiDB also permits genome improvements by capturing expert knowledge through the User Comments system and the Apollo genome annotation editor for structural and functional gene curation. FungiDB facilitates data exploration and analysis and contributes to advancing research efforts by capturing expert knowledge for fungal and oomycete species.

## Introduction

FungiDB (https://fungidb.org) is a component of the Eukaryotic Pathogen, Vector and Host Informatics Resources (VEuPathDB; Stajich *et al*. 2012; Basenko *et al*. 2018; Alvarez-Jarreta *et al*. 2024). Supported by the US National Institutes of Allergy and Infectious Diseases (NIAID) and the Wellcome Trust UK, VEuPathDB (https://veupathdb.org) is recognized as a Global Core Biodata Resource (https://globalbiodata.org/what-we-do/global-core-biodata-resources/). VEuPathDB empowers users to browse, query, and mine genomic-scale datasets spanning a diverse range of organisms, including hosts, invertebrate vectors of human disease, eukaryotic microbes, pathogenic and nonpathogenic species, environmental and epidemiological studies (https://clinepidb.org/; Ruhamyankaka *et al*. 2020), as well as microbiomes (https://microbiomeDB.org; Oliveira *et al*. 2018).

FungiDB supports different data types, including transcriptomics and coexpression datasets, proteomics, phenomics, single nucleotide polymorphisms (SNPs), and more. The search strategy system, enriched with Boolean operators, offers a versatile system for data mining and cross-species comparisons. Users can leverage different data types and test hypotheses via in silico experiments. The integrated VEuPathDB Galaxy supports custom data analysis and visualization (The Galaxy Community 2022). Users can also upload and access datasets in the “My Workspace” portal that supports private data analysis. Users can also easily contribute expert knowledge through the User Comments system and Apollo, a collaborative platform for genome annotation and curation (Dunn *et al*. 2019).

New data are regularly sourced from repositories such as the International Nucleotide Sequence Database Collaboration (Arita *et al*. 2021; https://www.insdc.org) and the Sequence Read Archive (Sayers *et al*. 2022; https://www.ncbi.nlm.nih.gov/sra), as well as directly from the community. Standardized workflows handle data processing, and users can access the results through dedicated searches, record pages (e.g. gene record pages), and the genome browser JBrowse (Buels *et al*. 2016).

In this study, we present an overview of new data, tools, and features integrated into FungiDB since FungiDB publication in 2018 (Release 37; Basenko *et al*. 2018). As of Release 66, FungiDB encompasses a total of 674 datasets, including over 319 annotated genome assemblies, including organisms on the World Health Organization's (WHO) fungal priority list (World Health Organization 2022). FungiDB also integrated 211 transcript expression studies, 13 ChIP-chip and ChIP-Seq studies, 84 genetic variation studies, 19 protein expression datasets, and more.

## New data, tools, and features in FungiDB

**Fig. 1.**
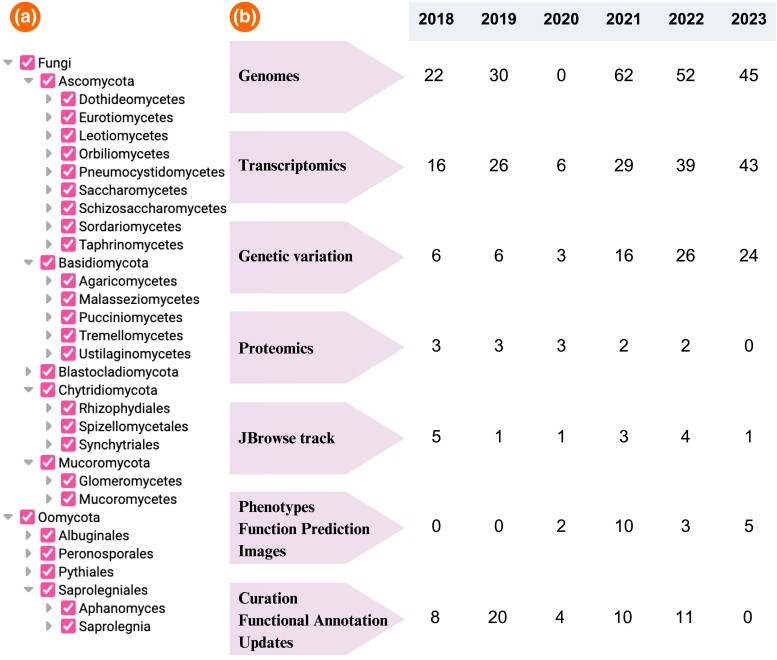
New data in FungiDB since FungiDB Release 37. a) The organism list in FungiDB demonstrates fungal and oomycete organisms currently represented in FungiDB. b) The newly integrated data are categorized based on data type. In 2020, the VEuPathDB platform successfully transitioned to a new database infrastructure that included a joint VEuPathDB-EBI pipeline for data processing. During this transition, dataset integration pipelines were temporarily suspended. New datasets are integrated on a bi-monthly database release cycle. Infrastructure development that supports new tools and data types is carried out continuously throughout the year.

FungiDB provides support for a wide array of model, nonpathogenic, and pathogenic organisms spanning the fungal and oomycete kingdoms (Fig. 1a). Human pathogenic fungi include those on the WHO fungal priority list, such as organisms in the critical, high-, and medium-priority groups (e.g. *Candida auris*, *Cryptococcus neoformans*, *Aspergillus fumigatus*, *C. albicans*, etc.; World Health Organization 2022). Since the last FungiDB publication in 2018, FungiDB has added nearly 500 datasets. Figure 1b provides an overview of the datasets incorporated into FungiDB from Release 37 (April 2018) to Release 66 (November 2023). These datasets contain published and unpublished data, manually curated data, records from external databases, community projects, genome reannotation initiatives, and more. Genome datasets are represented by new genome assemblies (with and without annotation) and genome replacements. The Transcriptomics category is represented by RNA-Sequencing, DNA microarray, and coexpression studies. The Genetic variation category provides access to SNP data calculated from whole-genome sequencing (WGS) projects. Proteomics datasets include mass spectrometry (MS) datasets and posttranslational modification data. JBrowse tracks include ChiP-Seq, Chip-ChIP data, user-provided annotations of transposable elements, or new gene models from genome reannotation projects. FungiDB also includes community-submitted data (images and phenotypes), records from other databases, and gene clustering data. Manual curation of selected genomes was performed from user comments and community-submitted files or downloaded from external databases.

**Fig. 2.**
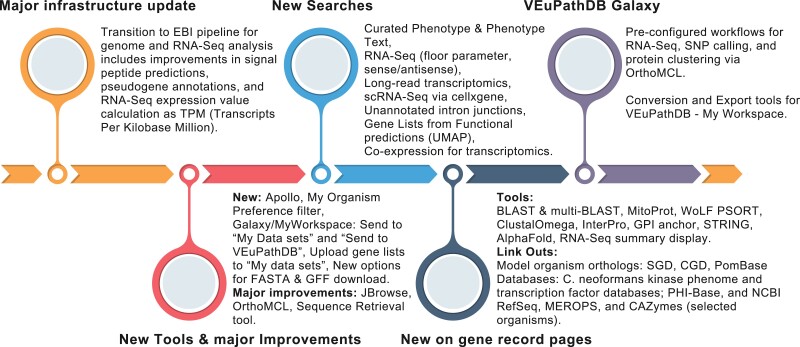
An overview of new features in FungiDB. New features are grouped into 5 categories: major infrastructure updates, new tools and significant improvements, new searches that facilitate data mining of new or established data types, updates to gene record pages, and the integrated VEuPathDB Galaxy–My Workspace platform in FungiDB.

FungiDB is regularly improving its resources to facilitate comprehensive data analysis by upgrading the infrastructure, visualization, and display interface. Figure 2 provides an overview of the new features and tools that have received significant updates or have been newly developed in FungiDB since Release 37 to facilitate comprehensive data analysis. Some tools are available directly from the Tools menu, while others are embedded within gene record pages. Some of these tools and new data are highlighted in greater detail below.

### Host–pathogen datasets

The VEuPathDB project supports data mining of dual, host–pathogen datasets. The fungal component of these studies can be accessed on FungiDB, while the host data can be accessed on HostDB (www.hostdb.org). For example, users can identify upregulated or downregulated genes and explore both host and fungal responses during infection caused by *C. auris*, *C. albicans*, *C. parapsilosis*, *C. tropicalis*, *A. fumigatus*, *Cr. neoformans*, and *Rhizopus delemar.*

### Phenotype evidence

FungiDB worked to integrate large-scale studies containing images and phenotype descriptions for several organisms. These data include the Neurospora Genome Project that systematically characterized the colony growth and morphology of *Neurospora crassa* knockout mutants (Colot *et al*. 2006; Dunlap *et al*. 2007). FungiDB also includes records from the high-throughput analysis of 10 different traits during hyphal growth, asexual sporulation, and the sexual cycle in *N. crassa* knockout mutants (Dunlap *et al*. 2007). Users can now explore these records within the dedicated phenotype query system. For example, users can identify genes based on knockout effects on ascospore number, perithecia morphology, and more.

In Release 48, 392 images from the COFUN transcription factor knockout project were integrated into the FungiDB gene record pages for *A. fumigatus* (Furukawa *et al*. 2020).

In Release 56, FungiDB incorporated a comprehensive functional genomic screening of *C. albicans* that compiled and normalized data from ∼400 phenotype screens (Gervais *et al*. 2021).

In Release 60, phenotypic annotations for 1,679 genes were integrated from MagnaGenes (v.2), a project that aimed to characterize gene function in the blast fungus *Pyricularia oryzae* (syn. *Magnaporthe oryzae*) (Foster et al. 2021). Phenotypes provided by MagnaGenes were mapped from controlled vocabularies to ontologies by the FungiDB team and integrated into the database.

FungiDB also processed curated phenotype records including virulence or drug resistance data for over 60 species from the Pathogen–Host Interactions database (PHI-Base; Urban *et al*. 2020, 2022; www.PHI-Base.org). In addition, FungiDB provides curated phenotypes for *Aspergillus* species, *Cr. neoformans*, *Fusarium graminearum*, and *P. oryzae* that were integrated over several release cycles from both in-house curation and external resources such as the Cryptococcus RO3 project (Inglis *et al*. 2014), the Aspergillus genome database (AspGD; Cerqueira *et al*. 2014), and the Candida genome database (http://www.candidagenome.org; Skrzypek *et al*. 2017).

### Copy number variation

The “copy number variation” (CNV) pipeline delivers 2 novel search mechanisms tailored to retrieve genes based on their copy numbers, as calculated using coverage from whole-genome DNA sequencing data. Users can apply the CNV search tool to sequences and genes. For instance, one way to discover regions of potential gains or losses in chromosomal segments is to employ the CNV query for genes. This query has 2 categories: Copy number and Copy number comparison. The Copy number search returns genes present at copy numbers (haploid number or gene dose) within a specified range. The Copy number comparison search compares the estimated copy number of a gene in the resequenced strain with the copy number in the reference annotation. The copy number in the reference annotation is calculated as the number of genes that are in the same ortholog group as the gene of interest and, therefore, are inferred to have risen from tandem duplication even in a common ancestor. Additionally, the Genomic Sequences by Copy number/ploidy search enables the identification of aneuploid chromosomes, and JBrowse tracks aid in the identification of segmental (partial chromosome) duplications.

### Function prediction

The Function Prediction tool includes the new “Identify Genes based on Gene Lists from Function Prediction” search. It allows users to access gene coexpression analysis data that can assist in the identification of gene clusters exhibiting similar expression patterns across various conditions. For *C. albicans*, the CalCENv1 coexpression network was projected with Uniform Manifold Approximation and Projection (O’Meara and O’Meara 2021), which identified 18 unique clusters. These clusters were integrated into FungiDB and can now be searched in FungiDB via the dedicated search.

### Structure analysis

AlphaFold DB (Varadi *et al*. 2022; https://alphafold.ebi.ac.uk/) is an open-access database that provides 3D protein structure prediction for over 200 million proteins. Users of FungiDB can take advantage of the imported AlphaFold predictions (Jumper *et al*. 2021) by accessing AlphaFold DB records and structure prediction visualization in the “Structure analysis” section on gene pages. Furthermore, with the “AlphaFold Prediction” search, users can identify genes that have structural predictions at AlphaFold DB.

### Transcriptomics search

When using the FungiDB RNA-Seq Evidence query, users can deploy several searches to identify differences in gene expression, using information sourced from transcriptomics datasets loaded into the database. In the case of stranded RNA-Seq data, users can now identify antisense regulatory transcripts by deploying the “sense and antisense expression” (SA) search. With the SA search, users can discover sense–antisense regulatory signatures.

Furthermore, users can identify oscillating genes in circadian transcriptomics datasets through MetaCycle query. The circadian rhythm data are analyzed via ARSER (Yang and Su 2010) or JTK_Cycle (Hughes *et al*. 2010), which are algorithms that extract and characterize rhythmic expression profiles of genes.

Coexpression datasets are now offered for *A. niger*, *C. albicans*, and *N. crassa*. For *A. niger*, the datasets were curated by the authors, who analyzed hundreds of microarray and RNA-Seq datasets (Schäpe *et al*. 2019, 2023) The *C. albicans* coexpression Network v1.0.0 (CalCENv1) includes an analysis of 853 RNA-Sequencing runs (O’Meara and O’Meara 2021), while the *N. crassa* coexpression network uses gene expression data from cells grown on different carbon sources (Wu *et al*. 2020). The coexpression query consists of “Identify Genes based on RNA-Seq Evidence” and “Identify Genes based on Microarray Evidence” searches, offering customizable search parameters (correlation, Spearman coefficient values, or highest reciprocal rank network distance).

### Unannotated intron junctions

The new “Unannotated Intron Junctions” search facilitates the discovery of unannotated or incorrectly annotated gene models. Genes returned by this search may be incompletely or inaccurately annotated due to missing introns/exons and/or alternative splice variants. Users can then correct these gene models by navigating to the genome editor Apollo (Dunn *et al*. 2019). The “Unannotated Intron Junctions” search can be found under the “Searches” menu and in the “Genes” > “Function Prediction” category.

### Tools for capturing expert knowledge from the community

FungiDB provides tools for data mining and display and for capturing expert knowledge from user-provided files or community projects. The User Comments system and Apollo (Dunn *et al*. 2019) are methods for capturing and improving structural and functional genome annotation in real time.

The User Comments system serves as a rapid data integration system for sharing the latest insights regarding genes. This system can be accessed directly from gene pages. User comments allow the incorporation of new information regarding gene function, mutant viability or growth phenotypes, updates to gene names, and other relevant details (e.g. PubMedIDs). Users can submit comments for a single gene or in bulk.

**Fig. 3.**
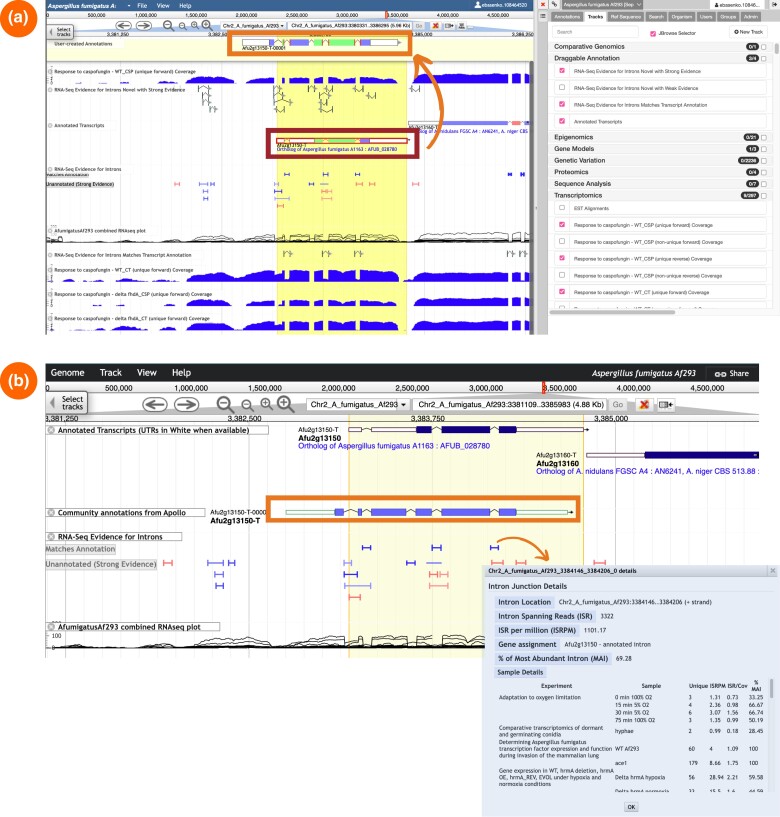
Components of the genome browser JBrowse display. a) The Apollo genome editor interface. Users can update gene models in the Apollo editor using the underlying data in FungiDB and draggable tracks (indicated by the red box). The revised and completed gene model shown in the “User-created Annotation” editor panel at the top (square box) can be visualized in JBrowse. b) A JBrowse display showing the new gene annotation from Apollo shown within the “Community annotations from Apollo” track (orange box). A pop-up window for the “RNA-Seq Evidence for Introns” track summarizes intron evidence generated from the integrated transcriptomes.

Apollo supports real-time manual curation of gene models, making it particularly well-suited for community-driven projects aimed at improving genome annotation quality. Using public or private RNA-Seq evidence, users can edit annotated genes (e.g. split, merge, or delete genes), annotate new, previously unidentified genes, and add or revise untranslated regions, exon, or intron boundaries. Apollo uses a JBrowse plugin and provides access to the data integrated into FungiDB (Fig. 3a), offering several tracks to aid in the manual gene curation process. For instance, users can drag and drop current gene models and intron tracks into the “User-created Annotations” workspace located at the top of the editor interface. Completed annotations are subsequently displayed as the “Community annotations from Apollo” track in JBrowse (Fig. 3b).

### Genome browser

The JBrowse genome browser is a platform for visualizing genomes and functional data, in addition to displaying updated gene models from Apollo. JBrowse includes several new developments: the “RNA-Seq Evidence for Introns” tracks and color coding for pseudogenes, in addition to the new “Community annotation from Apollo” track (Fig. 3b).

The “RNA-Seq Evidence for Introns” track summarizes intron evidence that aligns with the existing annotation. It also provides a graphical representation for unannotated introns supported by the evidence derived from the integrated RNA-Seq datasets. The JBrowse interface provides an interactive display through which users can access gene-centric details and additional navigation features. For instance, selecting the intron evidence tracks will generate a pop-up window displaying intron junction details and summary statistics calculated from the supporting RNA-Seq evidence. The “Community annotation from Apollo” track showcases gene model revisions imported from community-submitted evidence in Apollo. JBrowse also supports evidence generated from the annotation of specific genome elements, reannotation efforts, and other types of evidence, such as tracks for displaying transposable elements in *Coccidioides posadasii* (Kirkland *et al*. 2018), alternative gene annotations for *Sporothrix schenckii* 1099-18 (Giosa *et al*. 2020), and *A. nidulans* (Eurofung; Wortman *et al*. 2009) or ribosome profiling in *C. albicans* (Mundodi *et al*. 2021).

### Integrated Galaxy workspace for data analysis

Galaxy is a versatile platform for computational biology that uses the Findable, Accessible, Interoperable, and Reusable (FAIR) principles of data analysis (The Galaxy Community 2022). It is an open-source platform that delivers a user-friendly, web-based interface for omics-scale data processing. The VEuPathDB Galaxy is a cloud-based workflow platform offered through the third-party service, Globus (Foster 2011).

VEuPathDB Galaxy–My Workspace is an integrated platform for omics-scale data analysis. It offers a comprehensive array of preloaded genomes from all VEuPathDB genomics sites. It provides preconfigured workflows modeled after standard VEuPathDB-European Bioinformatics Institute (EBI) data processing pipelines. Users can deploy SNP workflows, and ortholog mapping via OrthoMCL and RNA-Seq workflows can be applied to both stranded and unstranded datasets. ChIP-Seq workflow processes ChIP-Seq FASTQ files. The workflow aligns reads to the reference genome with BWA-MEM (Galaxy Version BWA:0.7.12; SAMTOOLS: 1.2; Li 2013), followed by peak calling using Model-based Analysis of ChIP-Seq (MACS2; Galaxy Version 2.1.0.20151222.0; Zhang *et al*. 2008). The identified peaks are converted into bigWig files that can be visualized in JBrowse.

Users can customize any preconfigured workflows via the workflow editor. Alternatively, users can also build workflows from the ground up using the tools available in VEuPathDB Galaxy. The VEuPathDB Galaxy tool panel provides export tools designed explicitly for transferring private user datasets from VEuPathDB Galaxy into My Workspace within FungiDB. This tool kit includes Gene list, RNA-Seq evidence, bigWig, and OrthoMCL export tools. Once imported into My Workspace, datasets can be further analyzed and enriched using the FungiDB interface, which offers various options across different data types.

### Functional and structural annotation updates

FungiDB aims to integrate the latest information for the supported genomes by updating functional and structural annotation records. Functional annotation attributes such as gene names/synonyms, product descriptions, and Gene Ontology (GO) annotations and the searches that utilize these records have been updated using records from external databases, user comments, and manual curation of the scientific literature. The updates were made for several major pathogen and model systems, including but not limited to *Aspergillus* (*A. clavatus*, *A. fischeri*, *A. fumigatus*, *A. nidulans*, *A. niger*, and *A. oryzae*), *Candida* (*C. albicans*, *C. auris*, *C. glabrata*, *C. parapsilosis*, *C. tropicalis*, and *Clavispora lusitaniae*), *Co. immitis*, *Cr. neoformans*, *N. crassa*, *P. oryzae*, *Schizosaccharomyces pombe*, *Saccharomyces cerevisiae*, and *Ustilago maydis.* The external resources from which these annotations were obtained from include PHI-Base.org (Urban *et al*. 2022), Cryptococcus RO3 project (Inglis *et al*. 2014), MagnaGenes v.2, *N. crassa* genome project (Dunlap *et al*. 2007), AspGD (Cerqueira *et al*. 2014), Saccharomyces Genome Database (https://www.yeastgenome.org; Wong *et al*. 2023), Candida Genome Database (Skrzypek *et al*. 2017), FgMutantDb (Baldwin *et al*. 2018), GO Consortium (The Gene Ontology Consortium *et al*. 2023), and MIPS (Mewes *et al*. 2002). The FungiDB team also worked to integrate 1,265 gene model changes into Apollo for the dimorphic fungus *Mucor lusitanicus* CBS 277.49.

## Data processing pipelines

FungiDB relies on the joint data processing framework administered by the VEuPathDB project and the EBI. Data integration processes involve data processing through standard or custom-built pipelines. The results of the analyses are seamlessly integrated into the database infrastructure, which provides an easy-to-use web-based interface. Users can explore these data records by deploying the site search or dedicated searches tailored to individual data types. Additionally, various tools are available for in-depth data mining and exploration.

### Genomic pipelines

Genomic pipelines at FungiDB involve the integration of genomic sequences in FASTA format and annotation data in GFF3 format. These data are sourced from well-known repositories such as GenBank (Sayers *et al*. 2022), European Nucleotide Archive (Thakur *et al*. 2023), and DNA Data Bank of Japan (Fukuda *et al*. 2021). The genomic records are further improved with additional pipelines and algorithms for predicting and annotating DNA, RNA, and sequences with features such as protein domains or repetitive elements. For example, users can access gene model biotypes (e.g. tRNA, rRNA, or lcRNA) or query for the presence of specific DNA or protein motifs (e.g. signal peptides or transmembrane domains).

### Transcriptomics pipeline

Much like genomic data, RNA-Seq raw data are retrieved from data repositories, and datasets undergo processing through the EBI RNA-Seq alignment pipeline. This pipeline employs Trimmomatic 0.39 (Bolger *et al*. 2014) for eliminating low-quality data, HISAT2 2.2.1 (Kim *et al*. 2019) for aligning sequences to a reference genome, and HTSeq-count 2.0.4 (Anders *et al*. 2015) counts to generate reads per gene. Transcript per kilobase million (TPM)-normalized data are then used by search queries and data visualization methods.

### SNP calling pipeline

The standard SNP calling pipeline is tailored for processing short-read sequencing data (e.g. Illumina). It utilizes Bowtie2 for read alignment to a reference genome (Langmead and Salzberg 2012), SAM tools for generating sorted BAM files (Danecek *et al*. 2021), and GATK for read realignment around indels (McKenna *et al*. 2010). SNPs and indels are detected using VarScan (Koboldt *et al*. 2009). Users can access the results of the SNP pipeline on gene record pages, in JBrowse, and through dedicated SNP queries.

### CNV pipeline

The CNV analysis is performed on genomes annotated to the chromosome level. Similar to the SNP pipeline, CNV analysis employs Bowtie2 for read alignment (Langmead and Salzberg 2012) and HTseq-count for read depth count estimation (Anders *et al*. 2015). Users can deduce the presence of gene or chromosomal duplications by contrasting the TPM values for individual genes or the median TPM values for individual chromosomes with the median value across the entire genome.

### Proteomics pipeline

Proteomics datasets are integrated from user-processed files. FungiDB accepts analyzed data from MS pipelines, including qualitative studies (i.e. identification of peptides and proteins), quantitative studies (global quantifications of proteins), and identification of posttranslational modifications. Pipelines are used to load data from user-provided tab-separated files. Peptide sequence data are aligned to stored protein sequences, are projected to genomic coordinates, and become visible as JBrowse tracks. Quantitative datasets are stored in the core database, allowing queries to find proteins with particular fold change values across conditions. For example, users can identify differentially expressed genes in a proteomics experiment by selecting the reference and comparator samples and defining fold change search criteria. PTM data can be visualized on respective gene record pages and queried to find proteins carrying certain types of modifications.

### Orthology and synteny pipeline

FungiDB employs the OrthoMCL algorithm (Fischer *et al*. 2011) to identify orthologs and paralogs within all annotated genomes. This process involves utilizing BLASTP, several normalization steps, and Markov clustering to categorize protein sequences into ortholog groups. Each protein is assigned to a unique OrthoMCL ortholog group number, which is linked to a dedicated record page on OrthoMCL.org (https://orthomcl.org/). In FungiDB, users can access orthology and synteny records on gene record pages and identify orthologs via dedicated queries (e.g. the “Transform into related records” search). The orthology-based function prediction is also employed to assign putative Enzyme Commission (EC) numbers.

Additional details about VEuPathDB Data Analysis Methods can be located on the FungiDB website under the “Data” menu or explored on the VEuPathDB GitHub repositories (https://github.com/VEuPathDB).

## Accessing records in FungiDB

### Homepage

**Fig. 4.**
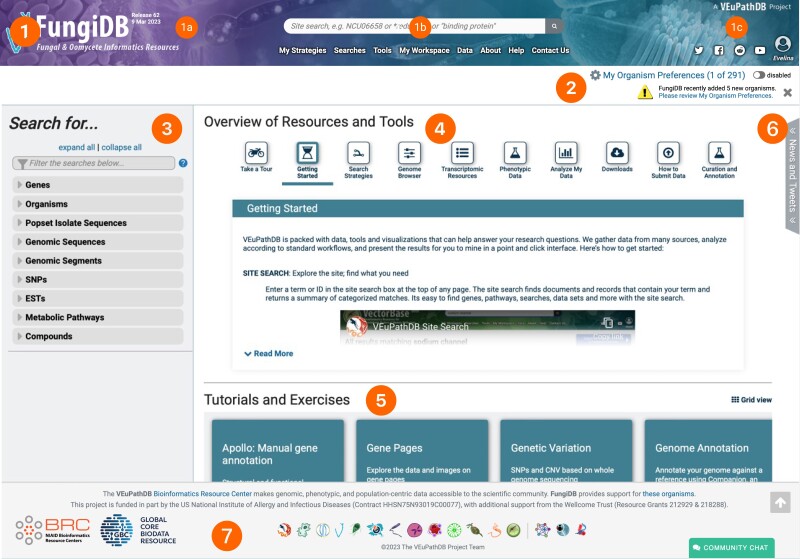
Core elements of the FungiDB homepage. 1) Banner: it includes 1a) release cycle information on the left, 1b) site search and menus centrally, providing access to nearly all searches, tools, and records in FungiDB, and 1c) account management and social media links on the right. 2) My Organism Preferences Filter: it is located directly below the banner and allows users to tailor database searches to a specified list of organisms. 3) Left “Search for…” panel: offers easy access to a categorized list of searches mirroring the options available in the “Searches” menu within the banner for consistent accessibility during site navigation. 4) Overview of Resources & Tools section: centrally located, it provides short tutorials on how to use FungiDB. 5) Tutorials and Exercises: positioned immediately under the Overview of Resource & Tools section. It offers comprehensive educational resources featuring in-depth overviews and exercises. 6) News & Tweets section: positioned on the right, this expandable section keeps users informed about new datasets, tools, features, and X (formerly Twitter) updates. 7) The footer: it contains links to other VEuPathDB databases and relevant resources. Additionally, there is a link to the community chat app where users can ask questions about how to use FungiDB resources.

The 7 core elements of the FungiDB homepage are depicted in Fig. 4. They are as follows: Banner, My Organisms Preferences Filter, “Search for …” Panel, Overview of Resources and Tools section, Tutorials and Exercises sections, News and Tweets, and footer. Collectively, these core elements work to provide an accessible, user-friendly interface, ensuring streamlined navigation and effective utilization of FungiDB's extensive array of features and resources.

### Site search

**Fig. 5.**
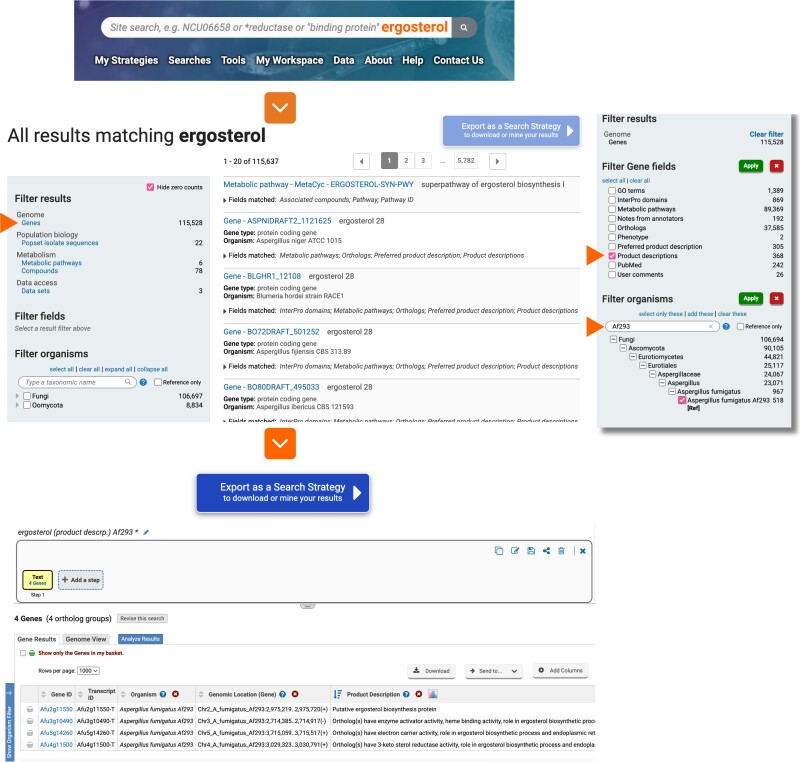
Site search, search categories, and data filtering. Searching for the term “ergosterol” yields a list of results. Gene results are subsequently refined with additional filters within the “Filter Gene Fields” and “Filter Organisms” to identify genes that contain “ergosterol” in the “Product descriptions” in the fungal organism *A. fumigatus* Af293. Users can export this search as a search strategy by activating the “Export as a Search Strategy” function. This function becomes activated as demonstrated by the dark blue color of the button when results are filtered on a single category (e.g. Genes).

The previous “Gene ID” and “Gene Text” searches in FungiDB were combined into a single feature, the site search. The site search is located centrally in the site header, and it allows users to query the database for a gene ID or a specific term (e.g. ergosterol; Fig. 5). The search results are organized by data type and record category (e.g. Genes, Metabolic pathways). Users can refine the output by applying filters specific to the chosen category. Organism-based filtering allows users to narrow results based on selected genomes from the taxonomy tree. Users can now also export search results as a search strategy using the “Export as a Search Strategy” button.

### Search strategy system and enrichment analysis

The updated strategy system provides a versatile collection of structured searches, allowing users to create unique, multistep in silico experiments. The available data-specific searches are categorized based on the data type they access. Users can initiate the search strategy from the site search as described previously or by using the “Search For…” Panel or the “Searches” menu within the banner. For instance, selecting searches under the “Genes” category will return a list of gene IDs based on specified parameters, while searches under the “Compounds” can yield Chemical Entities of Biological Interest compound IDs, metabolic pathways, and more.

The search strategy system is flexible and can be expanded by adding new steps or nested searches. These distinctive approaches to data mining, enhanced by Boolean operators, provide a comprehensive approach to data analysis.

**Fig. 6.**
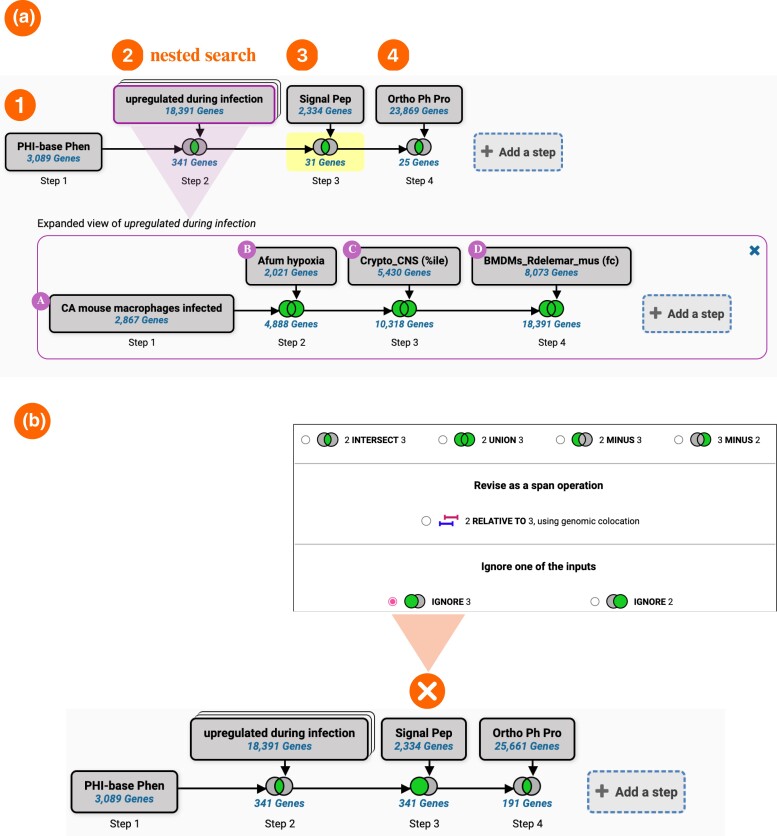
Creating multistep strategies in FungiDB. a) Example strategy identifying putative drug targets in several fungal species. Step 1: identify all genes manually annotated with a disease phenotype in PHI-Base. Step 2: nested RNA-Seq evidence search. The nested strategy consists of 4 steps to identify genes that are upregulated when: *C. albicans* is grown in the presence of macrophages A), *A. fumigatus* is exposed to hypoxia conditions B), *Cr. neoformans* is isolated from human cerebrospinal fluid C), and *R. delemar* is isolated from in vitro infections of mouse bone marrow–derived macrophages D). Step 3: cross-reference results from the nested search in step 2 with signal peptide prediction by SignalP. Step 4: identify genes that do not have orthologs in humans. This query utilizes the “intersect” Boolean operator, while the nested strategy combines all records from 4 species via the “union” operator. b) The “ignore” Boolean operators can remove steps from the strategy without deleting them. In this instance, the “ignore” Boolean operator is applied to exclude step 3 (SignalP prediction).

In Fig. 6, the FungiDB search interface was used to create a multistep search strategy to identify putative drug targets in several fungal species (*C. albicans* SC5314, *A. fumigatus* Af293, *Cr. neoformans* H99, and *R. delemar* RA 99-880). The search strategy is harnessing Phenotype, RNA-Seq, SignalP, and synteny and orthology records. It also uses a nested strategy approach to identify upregulated genes across multiple species using the integrated RNA-Seq evidence.

The modified strategy pinpoints genes annotated with disease phenotypes (step 1) that are also upregulated during infection in the 4 chosen species (step 2) and subsequently filters for genes without human orthologs (step 4).

Strategies, such as those illustrated in Fig. 6, are composed of a series of steps that are linked together using Boolean operators. These operators can intersect, combine, or subtract similar records and cross-refer different data types. The Boolean operators now include the “Ignore one of the inputs” option, which is particularly useful when users wish to omit a step without erasing it from the strategy completely [see Fig. 6a compared with Fig. 6b where the strategy was adapted to exclude SignalP results (step 3)].

**Fig. 7.**
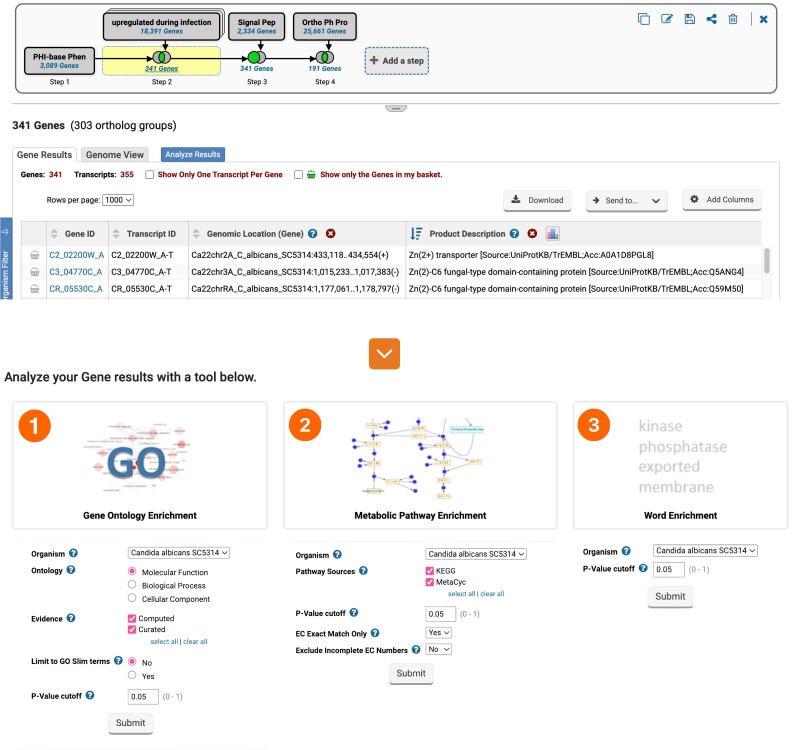
Key enrichment analysis tools. The enrichment tools are located under the “Analyze Results” tab, which is positioned above the results table. 1) GO Enrichment analysis enriches data on GO terms for molecular function, cellular component, and biological process annotations. 2) The Metabolic Pathway Enrichment relies on data from Kyoto Encyclopedia of Genes and Genomes (KEGG) and MetaCyc. This feature also allows the matching of results to precise EC numbers while excluding incomplete entries. 3) Word enrichment summarizes enriched product descriptions within a gene subset.

Search results can be further enriched by accessing options located under the “Analyze Results” tab (Fig. 7). The enrichment analysis leverages 3 essential tools: GO, Metabolic Pathway, and Word Enrichment. All 3 enrichment tools offer an adjustable *P*-value parameter. The GO and Metabolic Enrichment analysis tools also provide Fisher's exact test statistics, with multiple testing corrections via the Benjamini–Hochberg false discovery rate and the Bonferroni test.

### Gene pages

**Fig. 8.**
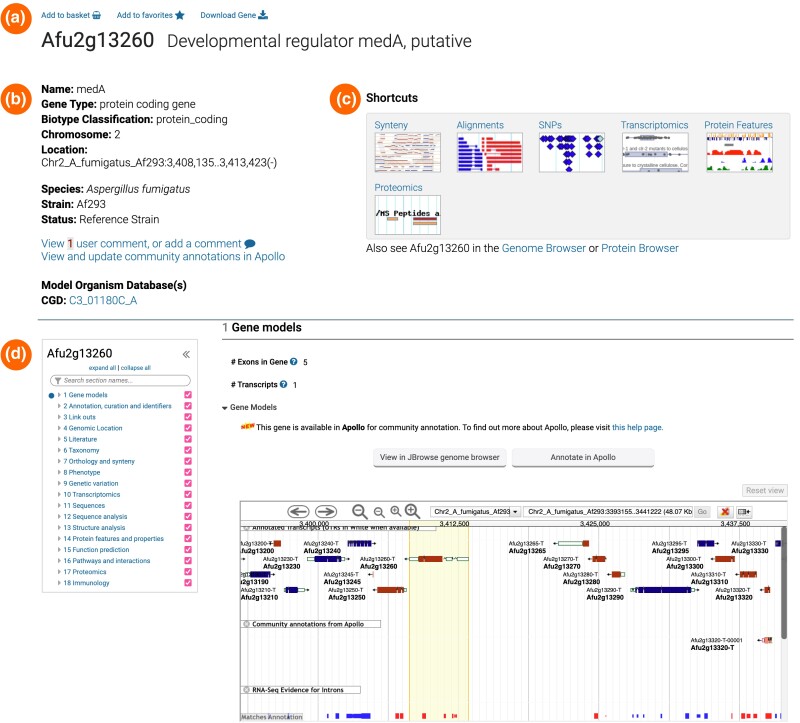
Components of the gene record pages. a) Links to export genes to baskets, add genes to favorites, or download gene data. b) FungiDB's organism summary, user comments, structural or functional updates from Apollo, and direct links to orthologs in prominent model databases. c) Shortcuts to the major gene record page section for easy navigation. d) Categorized content of the gene record page content.

Users can access categorized gene-specific data on gene pages (Fig. 8). With a mouse click from the top of the gene record page, genes can be stored in baskets or designated as favorites. Genes stored in baskets can be integrated into search queries as gene lists. Favorite genes are linked to a record dashboard where users can record specific information about the project, etc. Users can access their custom gene selections in the “My Workspace” section, which is accessible via the main navigation menu. Selecting the “Download Gene” option offers choices for downloading gene record–associated data, including text and columns from the gene records page, as well as FASTA, BED, and GFF files (Fig. 8a).

On gene pages, gene name, chromosomal position, and organism summary are presented alongside links to user comments, updates from Apollo, and links to orthologs in the model system databases (Fig. 8b). Changes and User comments submitted by the community are reflected on FungiDB record pages shortly after their submission. Both the User Comments system and Apollo require user authentication as they are associated with individual user accounts. Users can navigate gene record pages via shortcuts and the content menu system (Fig. 8, c and d). The combination of these tools allows for easy navigation between different content sections of the gene record pages.

## Enabling science and future perspectives

FungiDB is a knowledge base that supports fungal and oomycete research. The platform is dedicated to integrating omics-scale datasets and expert community knowledge and has established working relationships with numerous scientific communities to integrate a wide range of published and prepublication data.

FungiDB follows FAIR principles and ensures that all data analysis records are freely accessible and can be easily mined and interpreted using a suite of web-based bioinformatics tools. Researchers can scrutinize, export, and leverage a plethora of data types and can also compare and apply this analysis to their laboratory-based investigations and private data. The platform supports hundreds of genomes, is continuously updated and curated, and is widely cited in the primary literature.

Several new technologies have emerged such as single-cell RNA-Seq and long-read sequencing data for transcriptomics, which have the potential to significantly improve genome annotation, the understanding of gene expression, our knowledge of metabolic pathways, etc. Improved understanding of phenotypes can also be linked to metadata such as sample collection, geospatial distribution of strains with pathogenic potential, and clinical data. FungiDB plans to support these developments in a number of areas. Below are a few tools that are in development or near completion and soon will be accessible in FungiDB.

### Single-cell RNA-Seq query

The newly developed single-cell RNA-Seq functions will soon be available on FungiDB. Users will be able to visualize data directly on gene record pages or explore the data in greater detail in the CELLxGENE application (Li *et al*. 2020). This application offers UMAP output, normalized expression values, and histograms illustrating the distribution of normalized expression values for a particular gene across all cells, as well as the ability to perform simple analysis, such as differential expression. FungiDB plans to integrate single-cell data for *C. albicans* and *S. cerevisiae* starting in 2024.

### Pipeline for long-read data

A recently developed workflow for long-read RNA-Seq datasets uses minimap2 (Li 2018) and TALON (Wyman *et al*. 2020) to map reads, and call novel transcripts will soon be available on FungiDB (https://github.com/VEuPathDB/long-read-rnaseq-nextflow). The pipeline performs a clean-up of transcripts or noncanonical splice junctions unsupported by the reference annotation, and where possible, it also cleans reads by performing local realignment. TALON annotator collapses the alignments into novel transcripts and creates a database. Alignments and the GFF files generated from this pipeline will be loaded into JBrowse, aiding gene model evaluation. Queries will be available to find genes where models predicted from long-read data do not match the annotation and to find regions of the genome containing evidence for unannotated genes.

### Secondary metabolites pipeline

FungiDB already offers an InterPro search that identifies genes based on the presence of protein domains, but this is of limited utility when identifying secondary metabolites genes with complex criteria for domain presence (e.g. nonribosomal peptide synthetases genes are characterized by the presence of at least 3 domains: AMP-binding (PF00501), PP-binding (PF00550), and Condensation (PF00668; Kjærbølling *et al*. 2020). More complex searches are possible using the Strategies system, and several public searches have been made available as examples. We are working to create a dedicated search using InterPro domains as a basis for identifying members of known secondary metabolite gene clusters. We are also evaluating antiSMASH for incorporation into our analysis pipeline (Blin *et al*. 2023).

### MapVEu

The VEuPathDB's MapVEu web application and the exploratory data analysis platform provide a convenient interface for accessing and examining geospatial data linked to pathogen and clinical information collected worldwide. Within MapVEu, users can tailor maps according to organism clades, genetic markers, GPS coordinates of collections, and other parameters. Users can then visualize these data through a user-friendly and responsive interface that offers graphical plots that significantly improve the visualization and comprehensive analysis of the underlying data. Currently, this platform is accessible on the VectorBase.org website, which provides access to omics-scale datasets for human disease vectors such as mosquitos and ticks. FungiDB is exploring the MapVEu platform's capabilities and working to extend this functionality to fungal and oomycete pathogens. The first dataset for release will encompass *C. auris* isolate data from the WGS experiments integrated in FungiDB.

### Transferring genome annotations

FungiDB is testing an annotation transfer procedure for different genome assemblies for the same species. This method involves utilizing the liftoff program (Shumate and Salzberg 2021). Lifted annotations (GFF) will be displayed as alternative gene model annotations in a dedicated JBrowse track. We will also aim to provide liftoff statistics for passed and failed gene transfers, including information about internal stop codons, missing coding sequences, mismatches in protein sequences, and more.

To learn more about datasets we are working on, users are invited to explore information provided under the “Data” > “Data sets we are working on” page on FungiDB.

## Outreach

FungiDB actively participates in major conferences and meetings and engages with the scientific community by fostering communication with data providers and database users. FungiDB also aims to understand the community' needs by gathering feature and tool suggestions and dataset nominations. Users can contact FungiDB through the help desk email (help@fungidb.org), social media platforms, or the Contact Us form (https://fungidb.org/fungidb/app/contact-us). FungiDB organizes virtual and in-person workshops, including the Fungal Pathogen Genomics workshop organized in collaboration with Wellcome Connecting Science and other web-based bioinformatics resources. FungiDB provides help desks at scientific conferences where users can learn more about FungiDB and connect with FungiDB staff. Numerous educational resources, such as webinars, tutorials, and workshop materials, are accessible through the “Learn how to use VEuPathDB” page (https://fungidb.org/fungidb/app/static-content/landing.html).

## Data Availability

FungiDB is accessible at https://fungidb.org. Most of the VEuPathDB source codes are available publicly on the GitHub repository (https://github.com/VEuPathDB). Users can access versioned database records from the “Download Data Files” page (https://fungidb.org/fungidb/app/downloads).
